# Adeno-Associated Viral Vectors as Versatile Tools for Parkinson’s Research, Both for Disease Modeling Purposes and for Therapeutic Uses

**DOI:** 10.3390/ijms22126389

**Published:** 2021-06-15

**Authors:** Ana Fajardo-Serrano, Alberto J. Rico, Elvira Roda, Adriana Honrubia, Sandra Arrieta, Goiaz Ariznabarreta, Julia Chocarro, Elena Lorenzo-Ramos, Alvaro Pejenaute, Alfonso Vázquez, José Luis Lanciego

**Affiliations:** 1 Centro de Investigación Médica Aplicada (CIMA), Department of Neurosciences, Universidad de Navarra, 31008 Pamplona, Spain; ajrico@unav.es (A.J.R.); eroda@unav.es (E.R.); ahonrubia@unav.es (A.H.); sarrietae@unav.es (S.A.); garizna@unav.es (G.A.); jchocarrog@unav.es (J.C.); elr@unav.es (E.L.-R.); apejenaute@unav.es (A.P.); 2 Centro de Investigación Biomédica en Red de Enfermedades Neurodegenerativas (CiberNed), 28031 Madrid, Spain; 3 Instituto de Investigación Sanitaria de Navarra (IdiSNA), 31008 Pamplona, Spain; alfonso.vazquez.miguez@cfnavarra.es; 4 Department of Neurosurgery, Servicio Navarro de Salud, Complejo Hospitalario de Navarra, 31008 Pamplona, Spain

**Keywords:** alpha-synuclein, animal model, neuroprotection, disease-modifying therapy, precision medicine

## Abstract

It is without any doubt that precision medicine therapeutic strategies targeting neurodegenerative disorders are currently witnessing the spectacular rise of newly designed approaches based on the use of viral vectors as Trojan horses for the controlled release of a given genetic payload. Among the different types of viral vectors, adeno-associated viruses (AAVs) rank as the ones most commonly used for the purposes of either disease modeling or for therapeutic strategies. Here, we reviewed the current literature dealing with the use of AAVs within the field of Parkinson’s disease with the aim to provide neuroscientists with the advice and background required when facing a choice on which AAV might be best suited for addressing a given experimental challenge. Accordingly, here we will be summarizing some insights on different AAV serotypes, and which would be the most appropriate AAV delivery route. Next, the use of AAVs for modeling synucleinopathies is highlighted, providing potential readers with a landscape view of ongoing pre-clinical and clinical initiatives pushing forward AAV-based therapeutic approaches for Parkinson’s disease and related synucleinopathies.

## 1. AAV Landscapes

Adeno-associated viral vectors (AAVs) are members of the *Parvoviridae* family and *Dependoparvovirus* genus. AAVs are non-pathogenic and replication-defective viruses, requiring a co-infection with adenovirus or herpes simplex virus to complete the replication cycle. In the absence of a helper virus, AAVs can produce a latent infection in which the viral genome persists, integrated in infected cells [1]. AAVs were first identified by electron microscopy as contaminants of adenoviral stocks produced in in vitro preparations [2]. AAVs are characterized by their non-enveloped icosahedral capsid carrying a 4.7 kilobase single-stranded linear DNA genome packaging that contains two open-reading frames encoding four non-structural proteins (Rep40, 52, 68, and 78), the capsid (Cap) gene encoding for three structural proteins (VP1, VP2, and VP3), and a small viral co-factor required for assembly known as the assembly-activating protein. The replication gene (Rep) encodes four proteins that are essential for replication, packaging, and transcriptional regulation, and it is the key component in establishing the site-specific integration. The genome is flanked by two 145 nucleotides inverted terminal repeats (ITRs) that fold into T-shaped hairpins, which are the only essential sequences required in cis for the replication and packaging of the AAV genome [3]. Although the viral capsid structure is preserved, different capsid surface domains and slight variations in the amino acid composition result in different AAV serotypes, with up to 12 natural serotypes (from AAV1 to AAV12) and more than 100 variants being described to date [4]. A preferential CNS tropism has been reported for AAV1, AAV2, AAV4, AAV5, AAV7, AAV8, AAV9, and AAVrh10 [5], where AAV9 and AAVrh10 ranked as the serotypes showing the highest CNS tropism (Tanguy et al., 2015; Karim Bey et al., 2020).

AAV vectors containing the Rep sequence have a preferential integration into the AAVS1 site (only present in humans and NHP) of Ch19, whereas most Rep-independent recombinant AAVs vectors will not include Rep and will not integrate specifically. AAVs are promising for treating a number of neurological disorders [6]. However, random integration may result in mutagenesis potential and oncogenesis [7]. Accordingly, for the production of a given recombinant AAV, the *Rep* and *Cap* genes of the wild-type AAV are replaced by the transgene of interest and the regulatory sequences required for the promoter and the poly A sequence [8,9,10]. It is worth noting that AAVs allow transduction of both dividing and non-dividing cells, affording a stable, long-term gene expression, maintained up to 8 years in non-human primates (NHPs) [11] and up to 10 years in humans [12]. The ability of AAVs for transducing post-mitotic cells such as neurons is an important characteristic when dealing with AAV use in neurodegenerative disorders, as these viral vectors currently represent the safest choice for gene therapy. Therapeutic approaches other than AAVs, such as small molecules, nanocarriers, and exosomes (among others) are currently under development, collectively also representing appealing choices that still remain in early developmental stages [13,14,15]. Nevertheless, the AAV field is currently in the spotlight, already entering the clinical field, and based on robust preclinical evidence. Indeed, AAV-based therapies afford a stable, long-term expression for a given gene, with a disease-modifying potential after one single AAV delivery.

There are two main approaches for gene transfer into the CNS, the in vivo and ex vivo strategies. In the ex vivo approach, neurons are genetically engineered to produce the therapeutic protein and then transplanted into the host. The in vivo approach involves the direct insertion of genetic material into the CNS, mainly through the injection of viruses containing the gene of interest [16]. For the purposes of this review, we will remain focused only on the use of AAVs for the in vivo approach.

When designing any given AAV-based experiment in the CNS field, a number of important items regarding the AAV structure itself need to be taken into consideration, namely the selection of the best suited AAV serotype together with the choice of the most convenient promoter (e.g., the sequence driving transgene expression). That said, it is also known that different AAV serotypes exhibit preferred cellular-specific tropism; therefore, the AAV serotype choice can further be tailored when targeting a given brain territory and indeed important differences can be observed between AAV deliveries in adult vs. neonatal specimens [17].

Promoters can broadly be categorized as either ubiquitous or cell-specific, i.e., ubiquitous promoters drive transgene transduction both in neurons and glial cells, whereas a broad portfolio exists for specific expression in neurons vs. different types of glial cells, as well as within a number of neuronal phenotypes [18]. Regarding ubiquitous promoters for CNS delivery, CAG, CBA, GUSB, and EF1α are the ones most commonly used [18]. CAG is a strong synthetic promoter frequently used in mammalian expression vectors composed by the cytomegalovirus early enhancer element (C), the promoter of the chicken beta-actin gene (A), and the splice acceptor of the rabbit beta-globin gene (G). CBA is the chicken beta-actin promoter; GUSB is the promoter of beta-glucuronidase. Finally, EF1α is a constitutive promoter of human elongation factor-1 alpha.

Although using an efficient promoter is obviously a pre-requisite for any successful gene therapy approach, it is worth noting that different promoters may have different potencies when driving transgene expression. Indeed, when considering clinical translation, the choice of a strong promoter might not be viewed as the best strategy, bearing in mind that undesired side effects such as toxicity phenomena driven by unwanted excessive transduction of the encoded gene may appear. In other words, within the pre-clinical stage of development, one should ensure appropriate selection of the weakest promoter driving sufficient transgene expression.

Moreover, interactions between AAV capsids and promoters driving cell type transduction regardless of virus permissivity have been recently reported in both rodent and NHPs, and these interactions include widely-used promoters such as CBA and JetI [19,20].

In summary, a balanced choice between the AAV serotype and promoter is required for a successful outcome of any gene therapy-based experiment. Moreover, and considering the limited cloning capacity of AAVs, the use of small-sized promoters is a convenient choice for leaving enough cargo space to accommodate large genes of interest, if needed [21,22].

## 2. Routes for AAV Delivery

Once a final choice is made on the selected AAV serotype and promoter, the next decision to be taken is which would be the most appropriate delivery route maximizing viral transduction within the desired area. It is worth stressing the fact that the choice of delivery route represents a critical issue in terms of safety and efficacy of the desired therapeutics. Besides delivery routes targeting neurosensory organs such as the eye or the cochlea, the most frequently used approaches for CNS applications can be broadly categorized into: (i) intraparenchymal, (ii) intra-CSF (intrathecal or lumbar administration, intracisternal and intracerebroventricular), and (iii) intravenous (Hudry and Vandenberghe, 2019). Moreover, the choice of a given delivery route is also often disease-tailored or even customized by disease stages of progression (Table 1).

**Figure 1:**
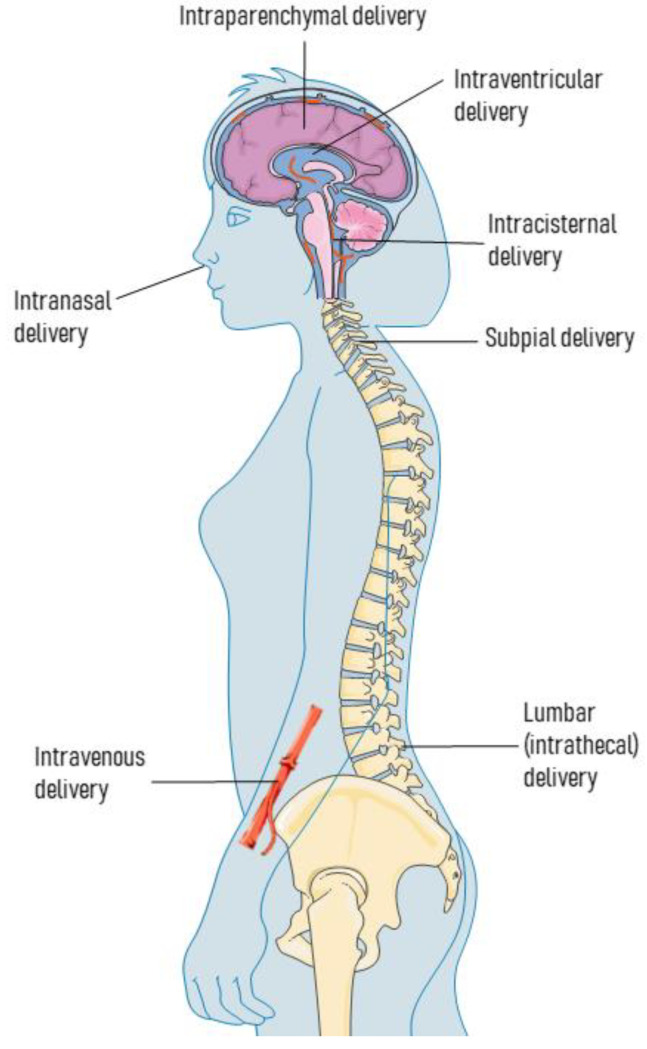
Most commonly used AAV delivery routes for PD. Although the intraparenchymal approach is by far the choice most often used, AAV deliveries can also be implemented through intra-CSF (intraventricular, intracisternal, and intrathecal), intravenous, and subpial routes.

The most commonly used routes for AAV deliveries within the context of Parkinson disease are the intraparenchymal, intra-CSF (lumbar, intracisternal, or intracerebroventricular), and intravenous. Other options less commonly used are either subpial [106,107] or intranasal [108]. The intranasal route of administration has often been considered for experimental therapies in the field of Parkinson’s disease (PD), other than AAV deliveries [13,14]. See Figure 1.

### 2.1. Intraparenchymal Deliveries

Intraparenchymal AAV delivery stands on stereotaxic surgery, a procedure where a needle or cannula is inserted directly in the desired target area, as defined with three coordinates (e.g., rostrocaudal, mediolateral, and dorsoventral coordinates). By delivering the viral vector this way, a high transduction efficiency is expected; therefore, the intraparenchymal delivery is the choice most frequently used in both pre-clinical and clinical studies (see Table 1 and Table 2) [109,110,111]. This route of administration offers a number of advantages, such as (i) maximizing viral concentrations into a specific brain area, (ii) no need for bypassing the blood brain barrier (BBB), (iii) broad cellular transduction expected even when infusing small volumes of viral particles, (iv) reduced immune response, and (v) very limited presence, if any, of off-target effects, i.e., transgene expression in peripheral body organs other than the brain. Reduced budgetary constraints also represent an additional advantage when compared to either intra-CSF or intravenous deliveries, where much larger volumes and higher viral titrations are required. Not surprisingly, the intraparenchymal delivery route has been appointed as the most popular choice for most of the currently ongoing clinical trials dealing with CNS neurodegenerative disorders, in particular for brain disorders where the initial pathological insult is limited to specific brain areas such as the substantia nigra in Parkinson’s disease (Table 2). When considering intraparenchymal AAV delivery, transduction levels in the brain are dependent on the (i) injected volume, (ii) rate of injection, (iii) viral titration, and (iv) affinity of a given AAV serotype for cell receptors at the injection site. For intraparenchymal deliveries targeting broad brain areas like the caudate-putamen nuclei, where maximizing the transduction area within the target structure is desired, the use of injection strategies such as pressurized convection-enhanced delivery (CED) [111,112,113] likely represents the most convenient choice.

It is also worth noting that the gene therapy field is a quickly changing scenario, with new arrivals being incorporated at a breath-taking speed, in particular those related to the new viral capsid variants designed for the retrograde spread of the AAV particles through brain circuits, ultimately transducing neurons from distant territories innervating the injection site. Although a number of AAV serotypes exhibit some inherent degree of retrograde spread, AAV6 and AAV9 in particular [61,128], newly-designed AAV capsid variants such as AAV2-retro [129], AAV-TT [82], AAV2-HBKO [83], and “barcoded” AAV-MNM004 and AAV-MNM008 [84] enable widespread transgene expression through retrograde transport. AAV2-retro and AAV2-HBKO have been successfully tested in both rodents and non-human primates [50,81,83], whereas AAV-TT, AAV-MNM004, and AAV-MNM008 have been so far only tested in rodents [82,84]. These AAV capsid variants share the option of transducing multiple brain areas with just one AAV deposit; therefore, they are very much appealing for the development of gene therapy treatments for neurodegenerative disorders characterized by the widespread aggregation of misfolded proteins. A similar rationale also applies when using retrogradely spreading AAVs for the purpose of modeling disseminated neurodegenerative proteinopathies in experimental animals. In this regard, the choice of the area best suited for retro-AAV delivery is dependent on the neurodegenerative disorder to be modeled.

### 2.2. Intra-CSF Deliveries

**Figure 2:**
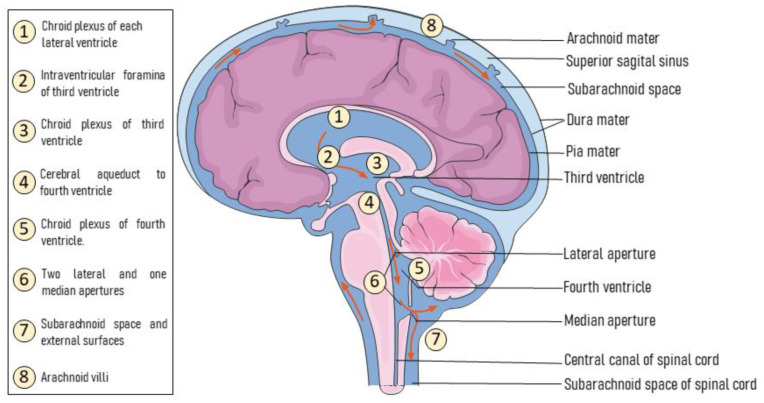
Schematic representation of CSF circulation. CSF is firstly secreted by choroid plexuses in each lateral ventricle (1) flowing through the interventricular Monro’s foramen into the third ventricle, where choroid plexuses (3) also add more CSF. From the third ventricle, the CSF flows through the cerebral aqueduct (4) down to the fourth ventricle, where more CSF is also added (5). Next, CSF leaves the ventricular system through two lateral apertures and one median aperture, known as foramina of Luschka and Magendie, respectively (6). CSF fills the subarachnoid space covering the external surfaces of the brain and spinal cord (7). At arachnoid villi, CSF is reabsorbed into peripheral venous blood through dural venous sinuses (8).

When considering AAV deliveries within CSF spaces, the distribution of viral particles will depend on the CSF circulation patterns, thereby raising some concerns about the inter-subject variability of intra-CSF deliveries. CSF is produced by choroid plexuses through brain ventricles, circulating through the ventricular system and finally reaching the subarachnoid space from where it is drained to the peripheral blood circulation [130] (Figure 2). CSF is produced continuously, being fully replaced up to five times per day.

Intra-CSF AAV deliveries collectively represent another feasible way for viral vector administration. This administration route bypasses the BBB, and it is less invasive than the intraparenchymal delivery (e.g., for intra-CSF deliveries other than the lateral ventricles, there is no need for stereotaxic surgery). Furthermore, when compared to intravenous administration, intra-CSF deliveries are expected to induce a lower immune response in peripheral organs. It can be performed either through lumbar puncture (e.g., intrathecal), the cisterna magna, or into the lateral cerebral ventricles [131]. Both AAV2.5 and AAV9 serotypes are able to achieve cellular transduction in the brain and spinal cord parenchyma following an intrathecal injection [86], and the same holds true for AAV-PHP.B [99] and AAVrh10 [96]. Although this delivery route has its own inherent advantages, vector dilution and the limited penetration/transduction of deep brain structures (e.g., poor diffusion from ventricles and subarachnoid space) are both limiting factors that need to be properly balanced before pushing forward intra-CSF deliveries for the purposes of animal modeling and/or disease interventions with a gene therapy therapeutic candidate. Intrathecal access to the subarachnoid CSF is achieved in routine clinical practice. Although less commonly used in daily clinical practice, access to the cisterna magna though an intracisternal approach instead of lumbar puncture can be 10 to 100-fold more efficient for brain transduction [95], despite a potential risk of damaging the medulla oblongata. In studies conducted in dogs, where intracerebroventricular and intracisternal delivery routes were compared, a similar transduction efficacy has been observed; however, encephalitis was reported as an unwanted event in animals after intracerebroventricular administration [131].

### 2.3. Intravenous Deliveries

The case of intravenous AAV administration is viewed as a relatively simple procedure, and much less invasive; however, it is also less effective for brain transduction as a result of the limited BBB passage for most AAV serotypes. Furthermore, when AAVs are delivered this way, a number of unwanted issues need to be taken into consideration, such as off-target effects, associated immune response, and potential toxicity phenomena. Although it is not an intrinsic feature of AAVs, some viral vector serotypes such as AAVrh10 [132] and AAV9 (the latter to a smaller extent, see [94,133,134]) can cross the BBB. Within the field of Parkinson’s disease, it seems that the limited penetration of the BBB by any given AAV serotype would result in a non-relevant therapeutic concentration ultimately reaching deep brain structures like the basal ganglia. In an attempt to overcome these limitations, an AAV9 variant known as AAV-PHP.B was introduced in 2016 [135], and was designed for more efficient BBB bypassing. It has been reported elsewhere that the improved penetration of the BBB by AAV-PHP.B is limited to the C57BL/6J mice strain [136], and the same likely applies to non-human primates [98]. Moreover, it is worth noting that BBB bypassing is more effective in neonatal rodent brains than in adults [137,138]. More recently, an improved version named AAV-PHP.eB has been released, showing a more efficient neuronal transduction in rats after intravenous administration [104,139]. At present, consensus exists on improved brain transduction of AAV-PHP.B variants compared to native AAV9 when injected into the cisterna magna in rodents [92]; however, the use of AAV-PHP.B in non-human primates is still a matter of debate. Moreover, the expression of the lymphocyte antigen-6 receptor (*Ly6a*) likely is the determining factor for the differential efficacy of AAV-PHP.B in transducing the CNS [140] across different mouse strains, as recently reported elsewhere [141]. *Ly6a* is almost exclusively expressed in brain endothelial cells, except for a minor expression in astrocytes and microglia, thus facilitating the BBB passage [140]. Interestingly, the presence of the *Ly6* locus in different mice strains is required for ensuring the transduction ability of some AAV serotypes [142]. Indeed, the lack of the *Ly6a* gene in NHPs likely explains the suboptimal performance of AAV-PHP.B in these animals [140,142,143].

### 2.4. Other Delivery Routes

Although technically very demanding, a subpial delivery route represents another feasible choice at hand. Subpial deliveries at cervical and/or lumbar spinal cord levels have been carried out in mice, pigs, and NHPs, showing efficient transduction of the spinal cord motor neurons [107]. When compared to the intrathecal administration of AAVs, the subpial approach seems to result in superior transgene expression. Bearing in mind that the length of the spinal cord in adult pigs of 35–40 kg of body weight is roughly similar to that of humans, a similar transduction would be expected [106].

Finally, the intranasal delivery route can also be considered as an alternative, less-invasive option. From the available AAV serotypes, AAV8 and AAV9 show the highest tropism for efficiently transducing olfactory sensory neurons [108,144]. In this regard, the intranasal approach has been suggested as an easy way for gaining direct CNS access, with an appealing potential for the treatment of neurodegenerative disorders [137].

When considering delivery routes all together, beyond the pros and cons stated above, two additional items merit extra attention. On the one hand, when compared to intraparenchymal deliveries, it is without doubt that intra-CSF and intravenous administrations require higher AAV titrations and volumes, something that can be viewed as an additional disadvantage particularly related to dose-escalation productions for clinical use. On the other hand, the presence of pre-existing neutralizing antibodies also represents a matter of concern, particularly regarding delivery routes other than intraparenchymal.

## 3. AAVs for Animal Modeling of Parkinson’s Disease

Although neurotoxin-based mammalian animal models of PD (6-OHDA and MPTP) have been instrumental in setting up the basis of our current understanding of basal ganglia function and dysfunction [145,146,147], these symptomatic models have failed to recapitulate the known neuropathology of this neurodegenerative disorder, where dopaminergic neurons die upon the progressive intracytoplasmic aggregation of misfolded alpha-synuclein (α-syn). Accordingly, the field is quickly moving forward towards the implementation of animal models of PD-like synucleinopathies in an attempt to lay the groundwork needed for the design and testing disease-modifying therapies [148].

### 3.1. Models Based on AAV-Mediated α-syn Overexpression

The discovery that α-syn is the major component of Lewy bodies [149] drastically changed the field of animal modeling in PD. Initial attempts were based on the development of several different murine transgenic mice lines overexpressing different forms of either mutated or wild-type α-syn. Although these transgenic mice lines are appealing choices for testing new therapeutic candidates targeting α-syn aggregation, in most cases these transgenic models lacked the appropriate phenotype and, indeed, neuronal loss in the substantia nigra pars compacta (SNpc) is often weak or even absent [150].

While transgenic mice have driven significant advances in the understanding of synucleinopathies, the use of viral vectors coding for α-syn have quickly become more popular in the field. These models stand on the use of viral vectors directly injected into the SNpc for transducing dopaminergic neurons with α-syn. From the available arsenal of viral vectors, both lentiviruses and AAVs have been the most often used choices [32,151]. The lentivirus-mediated transfer of α-syn resulted in dopaminergic (DA) neuronal loss in the SNpc after 6 weeks post-delivery [152]. Moreover, the use of AAVs overexpressing human A53T α-synuclein in rats resulted in the strong transduction of α-syn within TH+ neurons in the SNpc (above 90% of cells expressing α-syn), further inducing a variable degree of DA cell loss with different follow-up times [65,153,154,155]. Both AAV2 and AAV5 serotypes coding for α-syn have also been used in marmosets, achieving a well-established neuropathology together with a consistent DA cell loss ranging from 30 to 80% [146,151,153]. Quite a similar approach has been successfully tested in *Macaca fascilularis*, resulting in an up to 50% loss of TH+ neurons after a survival time of 17 weeks [156]. More recently, and by taking advantage of an AAV9 coding for A53T mutated α-syn, our group has reported a 39% loss of DA neurons in *Macaca fascicularis* 12 weeks post injection into the SNpc [157]. Although different choices made regarding AAV serotypes, promoters chosen, and ranges of survival times may account for the reported data dealing with the percentages reported above for α-syn induced DA cell death, it seems clear that the AAV-mediated enhancement of α-syn currently is in the spotlight in terms of development and validation of a new generation of animal models of synucleinopathy.

When using AAV coding for α-syn for the purposes of PD modeling, there are a number of issues that need to be taken into consideration. For instance, it is well known that marmosets harbor a naturally occurring A53T mutation in the SNCA gene, which could generate protection against A53T overexpression [72,148,158]. Next, although the A53T mutation is the one most broadly used, AAVs carrying mutations other than A53T, such as A30P and E36K, have also been successfully used in mice and marmosets [151,152]. Finally, it has been recently reported that the introduction of additional elements such as the woodchuck hepatitis virus post-transcriptional regulatory element (WPRE) enabled a greater nigrostriatal degeneration using AAV9 in mice [73] when compared with AAVs lacking WPRE.

### 3.2. Models Based on AAV-Mediated Neuromelanin Overexpression

Although animal models based on the AAV-mediated expression of α-syn collectively represent a major step forward in the field of PD and related synucleinopathies, it should be noted that these models share a common caveat, i.e., the obtained AAV-mediated expression of α-syn is well above what occurs naturally in physiological terms. In other words, the α-syn expression obtained this way is too high; therefore, although the loss of DA neurons is more progressive than neurotoxin-based models, it is quicker than desired, and DA neurons die too early from excessive α-syn production, i.e., before having a chance of aggregating α-syn in the form of Lewy bodies, as occurs in the human pathological conditions.

That said, it is worth recognizing that PD is a degenerative disorder only observed in humans, and animal species other than humans are not suffering from this disease. When thinking about what drives this specific susceptibility, the neuromelanin-related pigmentation of the SNpc in humans (the same applies to the locus cerouleus) is something unique, not shared with any other animal species; therefore, it can be hypothesized that neuromelanin accumulation may account for DA cell vulnerability. Indeed, neuromelanin aggregation in humans is a time-dependent phenomenon, something that needs to be taken into consideration bearing in mind that advanced age is the main known risk for PD. Only aged non-human primates exhibited some degree of pigmentation in the SNpc and locus cerouleus, although to an extent not comparable to that of the human SNpc. Such a physiological, time-dependent aggregation of neuromelanin in NHPs is preferentially observed in DA neurons located in the lateral and caudal regions of the SNpc, where the most vulnerable DA neurons are located (unpublished data).

While to what extent neuromelanin aggregation can be blamed for DA cell vulnerability is still an unresolved debate, a direct relationship between Lewy bodies and neuromelanin aggregates has been reported elsewhere [159]. Although this rationale (together with more evidence available in the literature remaining outside the focus of this review) can be taken as a smoking gun linking neuromelanin aggregation with PD pathophysiology, the most commonly used laboratory experimental animals lack neuromelanin, so such a potential link has been neglected so far in the field.

This scenario has completely changed very recently upon the design and validation of an AAV-based rodent model of PD characterized by neuromelanin aggregation [160]. These researchers have injected an AAV coding for human tyrosinase (the rate-limiting enzyme for neuromelanin) into the SNpc in rats. When injected this way, an age-dependent aggregation of neuromelanin is observed in the rat SNpc up to similar levels than those observed in elderly humans, together with a time-dependent PD-like symptomatic phenotype, nigrostriatal degeneration, and the presence of Lewy body-like aggregates. Although the mechanisms sustaining a link between neuromelanin production and α-syn accumulation are still poorly understood, a major advantage of this model when compared to AAV-mediated models of synucleinopathy is represented by the fact that the observed α-syn aggregation is physiological and driven by neuromelanin expression rather than directly induced by the injected AAV itself. In summary, this new model currently holds great promise and likely represents the animal model so far best mimicking the known pathophysiology of PD.

### 3.3. Other AAV-Based Models

Although models based on AAV-mediated α-syn overexpression are by far the ones most commonly used at present, a number of alternatives have been made available, including models related to leucine-rich repeated kinase 2 (LRRK2), and PTEN-induced kinase 1 (PINK1).

LRRK2 mutations are associated with autosomal dominant inheritance in familiar PD [161]. The generation of viral vector-mediated LRRK2 PD models using either adenoviruses or lentiviruses in rodents has been reported elsewhere [162,163]. LRRK2-based models failed to recapitulate several aspects of PD neuropathology, such as Lewy bodies and α-syn aggregates.

PTEN-induced kinase 1 (PINK1) is a serine/threonine-protein kinase. Knocking-down PINK1 with AAVs did not generated a significant dopaminergic cell death with a follow-up of up to 3 weeks in rodents [164]. Further research efforts will be needed to properly validate animal models based on the AAV-mediated administration of PINK1 interference mRNAs.

## 4. Use of AAV-Mediated Gene Transfer for Therapeutic Purposes

The use of AAVs for the treatment of CNS disorders exemplifies the translation of pre-clinical evidence towards clinical trials, beginning with pioneer experiences [8,165] up to a growing list of clinical trials (Table 2). Among the different AAV serotypes available, AAV2 and AAV9 ranked as the most commonly used within the context of PD (Table 2).

When considering PD under a simplistic view as a basal ganglia-related disorder primarily affecting the nigrostriatal pathway, the most rational scenario implies an intraparenchymal delivery route administering a given AAV either into the SNpc or in the striatum [166,167]. Although the SNpc is located very deeply in the brain and thus represents a demanding surgical target, its relatively small size makes the SNpc an affordable area to be covered by a direct intraparenchymal injection of an AAV. On the other hand, targeting the striatum is a more feasible surgical approach and indeed by going this way larger volumes of AAVs can be accommodated. Not surprisingly, most of the ongoing clinical trials are based on focused AAV deliveries targeting either the striatum or the SNpc (Table 2).

Gene therapy ongoing clinical trials for PD can broadly be categorized into (i) dopamine-related, (ii) neurotrophic factors, (iii) neurotransmitters and neuromodulation, and (iv) disease-specific mutations. Dopamine-related approaches take advantage of AAV coding for dopa-decarboxylase (AADC), the enzyme in charge of converting levodopa into dopamine. Early-stage clinical trials with AAV-AADC showed good safety and efficacy profiles, directly related to the percentage of the putaminal area transduced with the therapeutic vector [114,115,168,169], paving the ground for an ongoing phase II trial. However, AAV-mediated restoration of dopamine levels cannot be viewed as a disease-modifying strategy for PD. Quite the opposite applies to a number of gene therapy initiatives based on the use of AAVs as carriers of genes coding for neurotrophic factors such as glial cell-line neurotrophic factor (GDNF), and its close relative known as neurturin (NRTN). Both GDNF and NRTN exert a dopaminotrophic effect and are therefore likely good candidates for gene therapies. By taking advantage of intraputaminal AAV2-GDNF deliveries, disease progression was stabilized with a follow-up of 18 months, together with an enhanced uptake of the radiotracer F-Dopa [170] despite a somewhat limited transduced area of the putamen. Obtained endpoints using AAV2-NRTN instead of AAV2-GDNF under a similar approach showed less conclusive results [126], likely due to NRTN-increased expression restricted to the injection sites [171].

A completely different rationale underlies an AAV-based initiative for tuning down the hyperexcitability of the subthalamic nucleus (STN) that typically accounts in PD. With the purpose of switching the functional activity of the STN from excitation to inhibition, using AAV coding for the enzyme glutamic acid decarboxylase (GAD), [172]. Obtained results showed the safety of this approach, together with clinical improvement (measured by UPDRS motor scores) after 12 months of follow-ups. However, a subsequent phase II randomized trial failed to demonstrate motor improvement [121].

The demonstration that heterozygous mutations in the GBA1 gene (coding for a lysosomal enzyme known as glucocerebrosidase; GCase) are the main genetic risk factors for PD [173] has opened strong interest for increasing GCase activity as a potential disease-modifying treatment. Promising results obtained in pre-clinical studies carried out in mice and in NHPs [105,157,174] have paved the way for clinical translation, and in this regard it is worth noting that there is an ongoing clinical trial with PD patients carrying heterozygous GBA1 mutations through the intracisternal delivery of AAV9-GBA1.

## 5. Conclusions

It is without a doubt that we are at the beginning of a promising journey where gene therapy-based approaches will progressively gain appeal and popularity. Regarding the use of AAVs for the purposes of animal modeling, it is clear that any animal model has its own strengths and shortcomings. However, by taking advantage of the impressive versatility of AAVs at present we are closer than ever when adequately mimicking the underlying pathophysiology that typically characterizes PD. Furthermore, when coming to translational initiatives for disease-modifying purposes, both earlier and ongoing attempts have succeeded in demonstrating safety; although, efficacy needs to be improved. In this regard, finding a proper balance between the underlying rationale for a given approach and the most appropriate route for AAV deliveries are the key items ultimately driving clinical success. Within this scenario, it should be stressed that brain function and dysfunction specifically stand on neuronal circuits (e.g., neuronal circuits provide the brain with its unique identity and operational principles when compared to any other body organ); therefore, researchers and clinicians going this way should ensure they have a strong neuroanatomical background. Finally, it is worth noting that this is a quickly changing scenario where the best is surely yet to come.

## Figures and Tables

**Table 1:** Most commonly used AAV delivery routes in the context of Parkinson’s disease.

Delivery Routes	AAV Serotypes	Animal Species	References
Intraparenchymal	N.A.	Pig	[23]
	AAV1	Rat	[24]
	AAV2	Mouse	[25,26,27,28,29,30,31]
		Rat	[24,32,33,34,35,36,37,38,39,40,41,42,43,44,45,46,47,48]
		NHP *(M. fascicularis)*	[47,49]
		NHP *(M. mulatta)*	[50,51,52,53]
	AAV5	Mouse	[54,55,56]
		Rat	[24,32,57,58]
	AAV6	Mouse	[59]
		Rat	[60,61,62,63,64]
	AAV7	Mouse	[65]
		Rat	[65]
	AAV8	Rat	[61,66,67,68]
	AAV9	Mouse	[69,70,71,72,73]
		Rat	[61,72,74,75,76,77,78,79,80]
		NHP *(M. mulatta)*	[72]
	AAV2-retro	NHP *(M. mulatta)*	[50,81]
	AAV-TT	Mouse	[82]
		Rat	[82]
	AAV-HBKO	NHP *(M. mulatta)*	[83]
	AAV-MNM	Rat	[84]
Intra-CSF	AAV2.1	Mouse	[85]
	AAV2.5	NHP *(M. fascicularis)*	[86]
	AAV5	Mouse	[87]
	AAV6	Mouse	[88]
	AAV7	NHP *(M. fascicularis)*	[89]
	AAV8	Mouse	[87]
	AAV9	Mouse	[85,90,91]
		Rat	[92]
		NHP *(M. mulatta)*	[93,94]
		NHP *(M. fascicularis)*	[86,89,94,95,96]
	AAVDJ8	Mouse	[85]
	AAVrh10	Mouse	[91]
		Rat	[97]
		NHP *(M. fascicularis)*	[96]
	AAV-PHP.B	Mouse	[98,99]
		NHP *(M.mulatta)*	[98,99].
Intravenous	AAV9	Mouse	[100,101]
		NHP *(M. fasciularis)*	[94]
		NHP *(M. mulatta)*	[94]
	AAV-AS	Mouse	[102]
	AAV1-PHP.B	Mouse	[103]
	AAV-PHP.B	Mouse	[98,101,104,105]
		NHP *(M. mulatta)*	[98]
	AAV-PHP.eB	Mouse	[104]
	AAV-PHP.S	Mouse	[104]
Subpial	AAV9	Rat	[106]
		Pig	[106,107]
		NHP *(M. fasciularis)*	[107]

**Table 2:** Clinical trials for PD (http://www.genetherapynet.com/clinical-trials.html, last access 14/06/2021). AADC: human aromatic L-amino acid decarboxylase; NRTN: Neurturin; STN: Subtalamic Nucleus; IP: Intraparenchymal; IC: Intracisternal; CM: Cisterna Magna.

Clinical Trial Identifier	Duration	Phase	Gene	AAV Serotype	Delivery Routes	Region	Status	References
NCT01973543	2013–2020	I	AADC	AAV2	IP	Putamen	Completed	[114]
NCT02418598	2015–2018	I/II	AADC	AAV2	IP	Putamen	Terminated	[115]
NCT03065192	2017–2021	I	AADC	AAV2	IP	Putamen	Active, not recruiting	N.A. (*Neurocrine Biosciences)*.
NCT03562494	2018–2022	II	AADC	AAV2	IP	N.A.	Recruiting	[116]
NCT03733496	2018–2026	N.A.	AADC	AAV2	IP	Putamen	Enrolling, by invitation	[51,114,117]
NCT04167540	2020–2022	I	GDNF	AAV2	IP	Putamen	Recruiting	N.A. (*Brain Neurotherapy Bio, Inc.)*
NCT01621581	2013–2022	I	GDNF	AAV2	IP	Putamen	Active, not recruiting	[116,118,119,120]
NCT00643890	2008–2010	II	GAD	AAV2	IP	STN	Terminated	[121,122,123,124]
NCT00195143	2003–2005	I	GAD	AAV2	IP	STN	Completed	[122,125]
NCT01301573	2011–2012	N.A.	GAD	AAV2	IP	STN	Terminated	N.A. (*Neurologix, Inc.)*
NCT00252850	2005–2007	I	NRTN	AAV2	IP	Putamen	Completed	[126]
NCT00985517	2009–2017	I/II	NRTN	AAV2	IP	Putamen	Completed	[127]
NCT04127578	2020–2027	I/II	GBA1	AAV9	IC	CM	Recruiting	N.A. (*Prevail Therapeutics)*

## Data Availability

Data reported here are available from authors upon reasonable request.
